# Pancytopenia Concurrent With Metabolic Encephalopathy

**DOI:** 10.7759/cureus.67963

**Published:** 2024-08-27

**Authors:** Henry Zou, Akash Rau, Laura Thompson, David Henderson

**Affiliations:** 1 Family Medicine, Michigan State University College of Human Medicine, Grand Rapids, USA; 2 Family Medicine, Trinity Health Family Medicine, Grand Rapids, USA

**Keywords:** myelodysplastic syndrome, parvovirus b19, dehydration, metabolic encephalopathy, pancytopenia

## Abstract

Pancytopenia is a decrease in the number of cells in all peripheral blood cell lines and has been associated with anemias, cancers, chemotherapy, infections, and nutritional deficiencies. However, pancytopenia concurrent with encephalopathy is rare and not well-studied. We present a case of pancytopenia concurrent with metabolic encephalopathy. An 81-year-old female patient presented to the emergency department for two weeks of increased fatigue and hypersomnolence. The patient had trouble staying awake during the initial physical exam, and her laboratory results were significant for pancytopenia, hypercreatinemia, hypernatremia, hypermagnesemia, and alkalemia. She was admitted to the floor, diagnosed with metabolic encephalopathy and acute kidney injury, and treated with medication withholding, fluid resuscitation, and electrolyte repletion. She also received a comprehensive workup for pancytopenia, iron replacement, and red blood cell transfusion therapy. After her metabolic encephalopathy was resolved, she was discharged with plans to follow up with hematology/oncology for stable but unresolved pancytopenia. We hypothesize that the patient’s metabolic encephalopathy was likely due to acute kidney injury-induced uremia or dehydration. We further hypothesize that parvovirus B19 and myelodysplastic syndrome are possible etiologies for pancytopenia. Our case highlights the importance of closely monitoring patients taking Sodium-glucose co-transporter-2 (SGLT-2) inhibitors and loop diuretics for dehydration and subsequent organ failure.

## Introduction

Pancytopenia is a decrease in all three main blood cell types (red blood cells/RBCs, white blood cells/WBCs, and platelets) in the peripheral blood, and is a common incidental finding that indicates possible underlying disease processes affecting the bone marrow or peripheral cell lines [1]. Numerous etiologies can mediate pancytopenia, including aplastic and Fanconi anemia, multiple myeloma, myelodysplastic syndrome, autoimmune disorders, leukemia (acute and chronic), infections (cytomegalovirus, Epstein-Barr virus), antineoplastics, and nutritional deficiencies (vitamins B12, B9, and copper) [1]. However, pancytopenia concurrent with encephalopathy is a rare phenomenon that is not well-reported; previous case reports of this phenomenon have been associated with colchicine use [2], Leigh disease [3], and parvovirus B19 infection [4]. We present a case of pancytopenia concurrent with metabolic encephalopathy.

## Case presentation

An 81-year-old female patient with a history of chronic kidney disease, hypertension, myocardial infarction, diabetes, and obstructive sleep apnea presented to the emergency department (ED) with complaints of increased fatigue and altered mental status (the patient’s husband provided most of the history). The husband reported increased fatigue and hypersomnolence for the past two weeks since starting dapagliflozin for blood sugar control, noting that the patient sleeps for most of the day and has difficulty waking up to eat and drink. The patient also started bumetanide six weeks ago for fluid overload. The husband also reports darker, stronger-smelling urine for the past two weeks and worsening dementia over the past few months, which included tremors, confusion, and the inability to answer questions appropriately.

**Figure 1:**
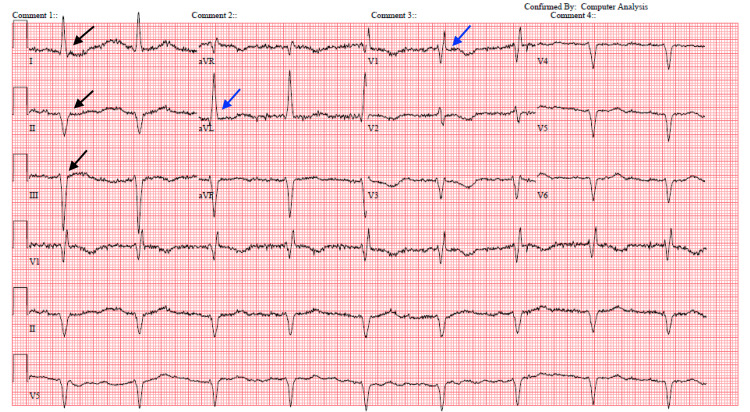
ECG showing sinus bradycardia Black arrows indicate the left anterior fascicular block. Blue arrows indicate the right bundle branch block.

On a physical exam, the patient required repeated verbal stimulation to stay awake and was alert and oriented to person and place, but not time. Despite limitations to the neurological exam due to the patient’s difficulty staying awake, her grip and plantar/dorsiflexion strength were 5/5 bilaterally, and light touch sensation was intact in all four extremities. Her complete blood count (CBC) was notable for pancytopenia (WBC 3.3, hemoglobin (Hgb) 9.8, platelets 112), her comprehensive metabolic panel (CMP) was notable for elevated creatinine (3.0 mg/dL), sodium (146 mEq/L), magnesium (2.8 mEq/L), and blood urea nitrogen (79 mg/dL), and her arterial blood gas (ABG) was notable for alkalemia consistent with contraction alkalosis (arterial pH = 7.48, bicarbonate = 36 mEq/L). Furthermore, an electrocardiogram (ECG) was notable for sinus bradycardia at 54 beats per minute (bpm), along with longstanding left anterior fascicular block and right bundle branch block (Figure 1).

**Figure 2:**
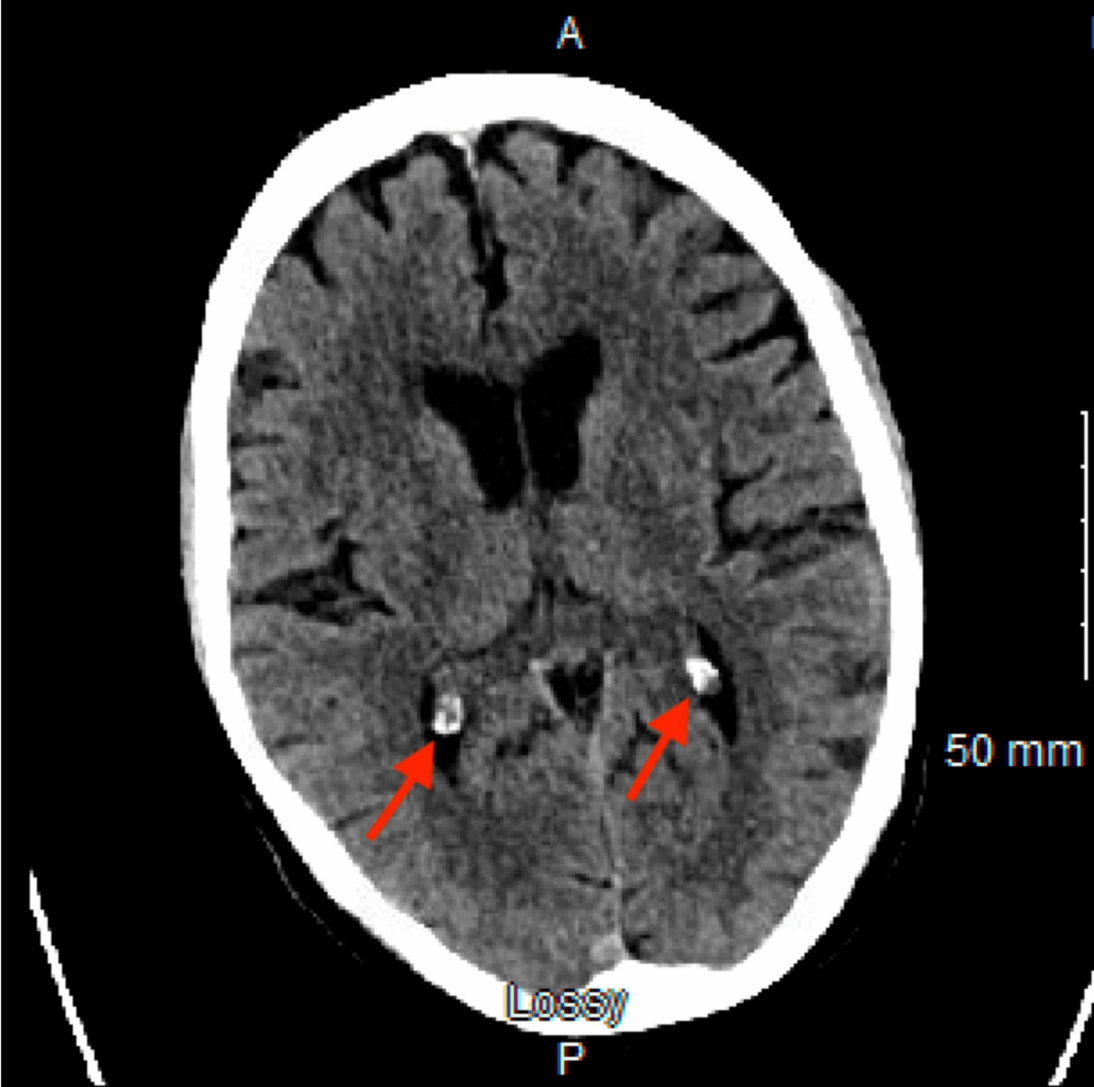
Unremarkable head CT without contrast Red arrows indicate calcification of the choroid plexus (normal aging process).

However, her head computed tomography (CT), urinalysis, thyroid stimulating hormone, and urine drug screens were unremarkable (Figure 2).

She was given one liter of intravenous (IV) normal saline in the ED. Given her altered mental status that precluded safe care at home, she was admitted to the floor on the same day. Over the next two days in the inpatient ward, she was diagnosed with metabolic encephalopathy, likely secondary to dehydration and decreased clearance of gabapentin and prerenal acute kidney injury (AKI). All diuretics (hydrochlorothiazide and bumetanide), potentially sedating medications (gabapentin, methocarbamol, and oxycodone), metoprolol, and dapagliflozin were held following consultation with nephrology. She was gently hydrated orally, and her electrolytes were monitored via basic metabolic panels (BMPs) and repleted as needed daily. 

On day three, an iron panel revealed low ferritin (18 ng/dL) and BMP revealed low potassium (2.9 mEq/L). Eight doses of intravenous (IV) sodium ferric gluconate complex 125 mg daily were initiated for her iron deficiency anemia.

By day four, her creatinine decreased to 2.4 mg/dL indicating improving renal function, but her pancytopenia worsened (WBC 2.6 K/mcL, Hgb 8 g/dL, platelets 77 K/mcL). The patient appeared more confused, followed commands intermittently, was not oriented to time or place, and had poor oral intake. Peripheral blood smear, ammonia, erythrocyte sedimentation rate (ESR), C-reactive protein (CRP), parvovirus B19, Epstein-Barr virus (EBV), cytomegalovirus (CMV), human immunodeficiency virus (HIV), vitamin B12 and B9, and methylmalonic acid (MMA) labs were ordered. Furthermore, hematology/oncology was consulted, who noted the patient’s history of breast cancer and ongoing tamoxifen treatment every six months as a risk factor for pancytopenia. IV D5 0.2% normal saline was initiated at 75 mL/hr to be weaned off as oral intake improved. Her haptoglobin levels (<8 mg/dL) and reticulocyte count (1.4) were low based on previous labs taken on the first day of hospitalization, indicating hemolysis, erythrocyte hypoproliferation, or possible liver disease given her elevated bilirubin (1.4 mg/dL) and AST (48 units/L) on day 6. 

By day five, her laboratory results were notable for mildly elevated CRP (1.0 mg/dL) and ammonia (110 mcmol/L), parvovirus B19 immunoglobulin (Ig)G and IgM antibodies, CMV IgG antibody, slightly elevated MMA, and positive direct Coombs test. However, her blood smear and vitamin B12 and B9 levels were normal. She received a transfusion of one unit of packed RBCs overnight; she was also started on lactulose 20 g twice daily for her hyperammonemia.

By day six, her husband reported that she had returned to her baseline mental status and resumed normal oral intake. Her AKI had resolved with creatinine decreased to 1.7 mg/dL; furthermore, her hypermagnesemia had resolved, and her hypernatremia and hypokalemia were improving (Table 1).

**Table 1:** Electrolyte trends over days 1-7 of hospitalization * = Abnormal value; -- = Data not collected; BUN: Blood urea nitrogen; EGFR: Estimated glomerular filtration rate; ALT: Alanine transaminase; AST: Aspartate aminotransferase.

Basic Metabolic Panel	Units	Reference Range	Day 1	Day 2	Day 3	Day 4	Day 5	Day 6	Day 7
Sodium	mmol/L	136-146	146*	--	146*	149*	144*	145*	145*
Potassium	mmol/L	3.5-5.0	3.4	--	2.9*	3.6	3.2	3.5	3.4
Chloride	mmol/L	95-105	98	--	105	110*	110*	110*	111*
CO_2_	mmol/L	23-29	21*	--	33	30	27	27	26
BUN	mg/dL	7-18	79*	--	67*	55*	43*	32*	29*
Creatinine	mg/dL	0.6-1.2	3.00*	--	2.40*	2.30*	1.70*	1.70*	1.60*
EGFR	mL/min/1.73m^2^	>90	15*	--	20*	21*	30*	30*	32*
Glucose	mg/dL	70-100	131*	106	79*	80	110	75*	74*
Calcium	mg/dL	8.4-10.2	10.6	--	9.6	9.7	9.4	9.6	9.5
Magnesium	mg/dL	1.5-2.0	2.8*	2.6*	2.5	2.4	2.2	2.1	2.1
ALT	unit/L	10-40	28	--	--	--	--	26	--
AST	unit/L	12-38	44*	--	--	--	--	48*	--
Bilirubin Total	mg/dL	0.1-1.0	1.8*	--	--	--	--	1.4*	--
Albumin	g/dL	3.5-5.5	3.3	--	--	--	--	2.8	--
Total Protein	g/dL	6.0-7.8	8.5*	--	--	--	--	6.7	--

**Figure 3:**
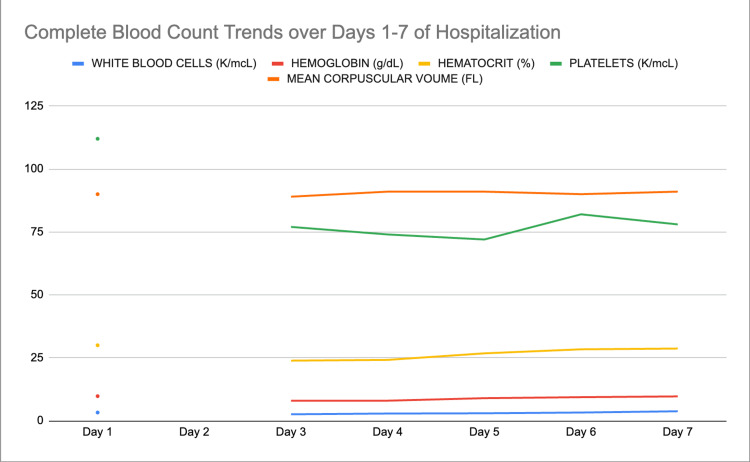
Line graph of complete blood count trends over days 1-7 of hospitalization

On day 7, her mentation and oral intake remained normal, and her hypokalemia resolved as well while her hypernatremia remained stable (Table 1). Her metabolic encephalopathy was considered resolved, and she was discharged home. However, her pancytopenia remained stable but unimproved (Table 2, Figure 3), and hematology/oncology agreed to continue monitoring in an outpatient setting.

**Table 2:** Complete blood count trends over days 1-7 of hospitalization * = Abnormal value; -- = Data not collected

Complete Blood Count	Units	Reference Range	Day 1	Day 2	Day 3	Day 4	Day 5	Day 6	Day 7
White blood cells	K/mcL	4.5-11.0	3.3*	--	2.6*	2.9*	3.0*	3.3*	3.8*
Hemoglobin	g/dL	12.0-16.0 (female)	9.8*	--	8.0*	8.0*	9.0*	9.4*	9.7*
Hematocrit	%	36-46 (female)	30.0*	--	23.9*	24.2*	26.8*	28.4*	28.7*
Platelets	K/mcL	150-400	112*	--	77*	74*	72*	82*	78*
Mean corpuscular volume (MCV)	FL	80-100	90.0	--	89.0	91.0	91.0	90.0	91.0

## Discussion

We hypothesize that parvovirus B19 infection and myelodysplastic syndrome are potential etiologies for our patient’s pancytopenia. Parvovirus B19 has been linked to pancytopenia, with a hypothesized pathophysiology being that increased viral-induced cytokines impair the regulation of phagocyte activity [5]. Our patient’s elevated IgG and IgM viral antibodies could indicate a recent parvovirus B19 infection. However, elevated IgG antibodies could be left over from a previous infection and may not be related to her current pancytopenia. The myelodysplastic syndrome has also been associated with pancytopenia, and cytostatic therapies such as tamoxifen have been identified as risk factors for secondary myelodysplastic syndrome [6]. Our patient has leukopenia and thrombocytopenia indicative of myelodysplastic syndrome and has several risk factors, including her advanced age and ongoing tamoxifen therapy [6].

If parvovirus B19 or myelodysplastic syndrome are the potential etiologies for pancytopenia, then the goal is to treat the underlying disease [1]. Treatments for myelodysplastic syndrome include supportive care, chemotherapy, or hematopoietic stem cell transplantation [1]. For parvovirus B19 treatments include transfusion, medication modification to improve immune function, and intravenous immunoglobulin [7]. 

Our patient received IV iron and a blood transfusion for anemia, lactulose for hyperammonemia, medication withholding, and electrolyte repletion. IV iron and blood transfusion were intended to improve anemia by increasing iron levels to stimulate erythropoiesis and replenish the RBC count; she responded positively to the transfusion, with her Hgb increasing from 8 to 9 g/dL. However, as the pancytopenia appeared stable and not severe enough to hinder the resolution of metabolic encephalopathy and electrolyte imbalances, our patient was deemed safe for discharge and outpatient follow-up with hematology/oncology.

Our patient’s metabolic encephalopathy was likely induced by dehydration, which is a well-documented adverse effect of dapagliflozin and other sodium-glucose cotransporter-2 (SGLT-2) inhibitors [8]. Dehydration encephalopathy describes central nervous system dysfunction precipitated by systemic dehydration; risk factors include advanced age (>68 years old), diuretic use, and systemic infection [9]. Given our patient’s advanced age, concomitant diuretic use, and potential parvovirus B19-induced pancytopenia, there were multiple risk factors that predisposed her to drug-induced dehydration encephalopathy.

## Conclusions

As more patients are treated concomitantly with SGLT-2 inhibitors such as dapagliflozin and loop diuretics for heart failure with reduced ejection fraction (HFrEF), clinicians should closely monitor for the development of severe dehydration during the initiation period and chronically. As experienced by our patient, uncontrolled dehydration can precipitate encephalopathy, acute kidney injury, and organ failure. Patients with multiple comorbidities, such as ours, should be monitored even more closely. While it is often convenient for the clinician to consider a unifying diagnosis for a patient presentation, this is a good example of a complex constellation of etiologies causing interplay and the ultimate patient presentation.
